# Identification of differentially expressed genes in chickens differing in muscle glycogen content and meat quality

**DOI:** 10.1186/1471-2164-12-112

**Published:** 2011-02-16

**Authors:** Vonick Sibut, Christelle Hennequet-Antier, Elisabeth Le Bihan-Duval, Sylvain Marthey, Michel J Duclos, Cécile Berri

**Affiliations:** 1 INRA UR83 Recherches Avicoles, Institut National de la Recherche Agronomique, F-37380 Nouzilly, France; 2 Institut Technique de l'Aviculture, Centre INRA de Tours, F-37380 Nouzilly, France; 3 INRA Centre de Ressources Biologiques des Animaux Domestiques et d'Intérêt Economique, Institut National de la Recherche Agronomique, F-78352 Jouy en Josas Cedex, France

## Abstract

**Background:**

The processing ability of poultry meat is highly related to its ultimate pH, the latter being mainly determined by the amount of glycogen in the muscle at death. The genetic determinism of glycogen and related meat quality traits has been established in the chicken but the molecular mechanisms involved in variations in these traits remain to be fully described. In this study, Chicken Genome Arrays (20 K) were used to compare muscle gene expression profiles of chickens from Fat (F) and Lean (L) lines that exhibited high and low muscle glycogen content, respectively, and of individuals exhibiting extremely high (G+) or low (G-) muscle glycogen content originating from the F_2 _cross between the Fat and Lean lines. Real-time RT-PCR was subsequently performed to validate the differential expression of genes either selected from the microarray analysis or whose function in regulating glycogen metabolism was well known.

**Results:**

Among the genes found to be expressed in chicken P. major muscle, 197 and 254 transcripts appeared to be differentially expressed on microarrays for the F vs. L and the G+ vs. G- comparisons, respectively. Some involved particularly in lipid and carbohydrate metabolism were selected for further validation studies by real-time RT-PCR. We confirmed that, as in mammals, the down-regulation of CEBPB and RGS2 coincides with a decrease in peripheral adiposity in the chicken, but these genes are also suggested to affect muscle glycogen turnover through their role in the cAMP-dependent signalling pathway. Several other genes were suggested to have roles in the regulation of glycogen storage in chicken muscle. PDK4 may act as a glycogen sensor in muscle, UGDH may compete for glycogen synthesis by using UDP-glucose for glucoronidation, and PRKAB1, PRKAG2, and PHKD may impact on glycogen turnover in muscle, through AMP-activated signalling pathways.

**Conclusions:**

This study is the first stage in the understanding of molecular mechanisms underlying variations in poultry meat quality. Large scale analyses are now required to validate the role of the genes identified and ultimately to find molecular markers that can be used for selection or to optimize rearing practices.

## Background

With changes similar to those that occurred in the pig industry, the poultry market is now characterized by increasing diversity of processed products [1]. As a consequence, poultry companies are now involved in food technology and product development, and improvement of meat processing ability has become a prevalent concern. As in pigs, post-mortem pH is a key factor controlling chicken meat quality [2]. Variations in ultimate meat pH (pHu) are responsible for variations in several breast meat properties, including water-holding capacity, colour and firmness [2,3]. Low ultimate pH results in "acid meat", with a pale aspect and reduced water-holding capacity [4], while high ultimate pH leads to DFD (dark, firm, dry) meat, dark in colour, with reduced shelf-life [5]. At the genetic level, there is a very strong negative correlation between the ultimate pH of breast meat and the level of muscle glycogen estimated by the glycolytic potential at the time of slaughter (rg -0.97) [3]. The glycolytic potential has also been shown to be highly heritable (h^2 ^0.43) [3]. Understanding the mechanisms and identifying the genes controlling muscle glycogen storage constitute a promising way to increase control of and improve chicken breast meat properties. It would make it possible to develop useful breeding tools, such as molecular markers, to select birds with expected meat properties, and help optimize rearing practices, *via *the study of gene regulation.

Glycogen is the main metabolic fuel for the anaerobic glycolysis which takes place post-mortem when muscles are no longer supplied with oxygen. The genetic control of muscle glycogen, and therefore meat quality, was evidenced first in mammals and more recently in the chicken [3]. In mammalian species, including the pig, mouse and human, a major gene, PRKAG3 which encodes the γ3 regulatory subunit of the AMP-activated protein kinase (AMPK), is responsible for variations in muscle glycogen content [6-10]. In the chicken, there is no information available suggesting that a major gene could be involved in the control of glycogen content in muscle. However, several studies have suggested that breast muscle glycogen content is related to growth and body composition: it decreases with growth rate and breast meat yield [2,11] and increases with carcass fatness [12,13].

The aim of the present study was to compare the expression profiles of muscles from chickens differing in muscle glycogen content and breast meat quality. In the first experiment, birds originating from two experimental lines (i.e. Fat (F) and Lean (L) lines) were compared. The F and L lines were originally divergently selected for and against the amount of abdominal fat [14] but they also exhibited differences in muscle glycogen content and in breast meat quality traits [13]. Despite a similar growth rate, the chickens from the F line were 3 times fattier than those from the L line. Moreover, due to high muscle glycogen content, the meat of the fat chickens exhibited a lower ultimate pH and higher drip loss and lightness than lean chickens. The differences in muscle glycogen between the F and L lines have been related to variations in mRNA encoding several enzymes regulating glycogen synthesis and degradation as well as in activation of AMPK by phosphorylation [13]. In the second experiment, the muscle transcriptomes were compared in individuals generated from the F_2 _population produced from these two lines (i.e. F_2_FL) and exhibiting extremely high (G+) or low (G-) muscle glycogen content. Individuals for this analysis were chosen according to their levels of glycogen in muscle, while differences in body fatness were much less pronounced than in the first model.

The use of a 20 K oligo microarray provided the first description of genes differentially expressed between breast muscles exhibiting high or low glycogen content, correlated with poor or high meat quality traits, respectively. This global approach was complemented by mRNA analyses on previously studied candidate genes [13] and on a subset of genes identified from array analysis. For genes with a human ortholog, further interpretation was based on the use of Ingenuity and Gene Ontology annotation databases highlighting several biological processes likely to be involved in muscle glycogen regulation.

## Results

### Carcass, muscle and meat quality traits of chickens used for gene expression analyses

The mean carcass and P. major muscle traits and SD are presented in Table 1. At 9 weeks of age, body weight was similar between F and L birds and between G+ and G- birds generated from the F_2_FL population. Breast meat yield was slightly higher in L than in F birds and similar between G+ and G-. Abdominal fat yield was 2-fold greater in F than in L birds while it was only 30% greater in G+ compared to G- birds. However, the muscle glycogen reserves were 61% higher in G+ than in G- birds while they were only 34% higher in F than in L birds. The ultimate pH of P. major muscle of F and G+, in relation to their greater glycogen content at the time of death, was higher than that of L and G- birds, respectively. The breast meat was lighter (greater L*) and less coloured (lower a* and b*), and exhibited more drip loss in F than in L birds. Only meat lightness (L*) was higher in G+ than in G- birds generated from the F_2_FL population.

**Table 1:** Body weights and yields, and *Pectoralis major *muscle and meat quality traits of animals used for expression analyses

	Fat	Lean		G+	G-	
**Chickens (n)**	**8**	**8**	***p value***	**8**	**8**	***p value***

*Growth and body composition*						
Body Weight (g)	1765 ± 99	1679 ± 178	NS	1891 ± 226	1979 ± 195	NS
Breast Yield (%)	12.4 ± 0.7	13.4 ± 0.8	< 0.05	12.0 ± 0.9	12.1 ± 0.7	NS
Abdominal Fat Yield (%)	5.2 ± 0.9	2.6 ± 0.7	< 0.001	3.8 ± 0.7	2.9 ± 0.8	< 0.05

*Breast meat quality traits*						
Glycolytic Potential (μM/g)	122 ± 8	91 ± 4	< 0.001	126 ± 9	78 ± 5	< 0.001
Ultimate pH	5.66 ± 0.06	5.86 ± 0.08	< 0.001	5.55 ± 0.07	5.88 ± 0.07	< 0.001
Lightness (L*)	49.7 ± 2.4	43.96 ± 2.1	< 0.001	50.6 ± 1.4	48.3 ± 1.7	< 0.01
Redness (a*)	-0.2 ± 0.6	1.7 ± 0.6	< 0.001	0.2 ± 0.7	0.4 ± 1.6	NS
Yellowness (b*)	11.0 ± 1.8	12.6 ± 0.9	< 0.05	11.2 ± 1.6	11.4 ± 2.0	NS
Drip Loss (%)	1.7 ± 0.7	1.0 ± 0.5	< 0.05	1.2 ± 0.65	1.1 ± 0.67	NS

The G+ and G- chickens were generated from the F_2_FL population produced from the 2 generation intercross between the founder Fat and Lean lines. The G- and G+ chickens correspond to the individual females exhibiting the lowest and the highest muscle glycogen content within the F_2_FL population. NS = non-significant.

### Differential analysis on microarray

Among the genes found to be expressed in chicken P. major muscle, 197 and 254 transcripts were differentially expressed between F and L and G+ and G-, respectively (Additional files 1 and 2). The gene expression fold-change ranged from 0.41 to 2.69 and 0.48 to 2.23 for the F vs. L and the G+ vs. G- comparisons, respectively. A trend was observed for a higher percentage of genes down-regulated in muscle with high glycogen content (57 and 60% in F and G+, respectively). Full details of gene name, function, accession number, fold-change and p-value for all differentially expressed transcripts are listed in additional files 1 and 2. Only 12 transcripts were recorded as differential in both analyses, i.e. F vs. L and G+ vs. G-, corresponding to 7 known genes (Additional file 3).

### Functional annotation

Among the genes that were reported to be differentially expressed between muscles with high or low glycogen content, 337 with a human ortholog were submitted to annotation analyses. When compiling the lists of genes expressed differentially between G+ and G- and between F and L chickens, the software Ingenuity Analysis Pathways 7.0 (IPA, Ingenuity System^®^, http://www.ingenuity.com) highlighted several biological functions (Table 2), including Lipid and Carbohydrate Metabolism. The genes associated with these two functions represent about 15% of the 337 genes considered for annotation analysis. When considering findings separately, IPA highlighted lipid metabolism and molecular transport as common pathways in the two models (G+/G- and F/L) and also specific biological functions for each of them: Cell Morphology, Cell Cycle, and Cell to Cell Signalling and Interaction for F/L, and Small Molecular Biochemistry, Cell Death, and Cellular Development for G+/G-. Gene Ontology (GO) terms are also widely used for global interpretation of the functions of genes revealed by differential microarray analysis. According to Gene Ontology, genes differentially expressed between F and L participated in several biological processes that can be grouped in 4 main biological functions: molecule transport and localization, and lipid, energy, and amino acid metabolism. Genes differentially expressed between G+ and G- belonged to biological processes especially related to lipid and energy metabolism, as well as developmental processes including cell growth, proliferation, differentiation and organization.

**Table 2:** Relevant biological functions identified from the annotation analysis

Category	P-value	Molecules
Lipid Metabolism	9.42E-05-2.56E-02	ABCA12, ABCA2, **ABHD5, ACSBG2, ACSL1**, **ADF**,**ADIPOR2**, ALDH1A1, **AOX1,CD38, CEBPB**,CETP**, CTSS**, DCI,DRD3,GOT2, **GRB10**,HMGCL, **HTT**,INSIG1, LASS4, LPAR1,**LPIN1,MTMR6**, MTMR7,PCTP, *PDK4*,**PHYH, PIK3CD**,PLA2G7, *PNPLA2*, **PRKAG2, PSAP**,RAB5A,**SGPL1, SLC27A1**,UCP3

Molecular Transport	9.42E-05-1.93E-02	ABCA12, ABCA2, **ABHD5, ACSL1, ADFP, ADIPOR2**,ALDH1A1, **ARNTL,CD38, CEBPB**,CETP, **CTSS**, DCI,DRD3**, F3, GHR**,GOT2,HCK, **HTT**,INSIG1, LASS4, LPAR1,**LPIN1**, NEB, P2RY2,PCTP, **PIK3CD**, PLN, *PNPLA2*, **PSAP,SGK1, SLC27A1**,TGFB2, TGFB3,**TRPC3**, UCN3, UCP3**, UGP2,VWF**

Small Molecule Biochemistry	9.42E-05-2.6E-02	ABCA12,ABCA2, **ABHD5, ACSBG2, ACSL1,ADAM10,ADFP,ADIPOR2**,ALDH1A1, **ALDH6A1, AOX1,CD38, CEBPB**,CETP**, CTSS**, DCI, DRD3,**FOXO3, GHR**,GLS,GOT2, **GRB10**,HMGCL,**HTT**,INSIG1, LASS4, LPAR1,**MTMR6**,MTMR7, NUDT3,PCTP, *PDK4*, **PIK3CD**,PLA2G7, *PNPLA2*,LPIN1**, PHYH**, **PRKAG2**,PRPS1, **PRPS2, PSAP**,RAB5A, **RPIA,SGPL1, SLC27A1**, TGFB2,TGFB3, UCN3, UCP3,**UGDH,UGP2**

Carbohydrate Metabolism	3.36E-04-2.6E-02	**ABHD5, ADAM10, ADIPOR2,ALDH2, CEBPB**,CETP, **FOXO3, GHR**, **HTT,IMPA2,MTMR6**, MTMR7,PCTP, *PDK4*, **PIK3CD**,*PLA2G7*, **PRKAG2, PSAP**,RAB5A, **RPIA,SOCS3**, TGFB2, TGFB3,UCN3, UCP3,**UGDH, UGP2**

Cell Death	3.36E-04 - 2.64E-02	ALDH1A1, **ATPA1**, BAG3, CD99, CDK2AP1, **CEBPB, CTSS, DAPK1, DCN, FGF1**, FGFR2, **FOXO3, GHR, HTT**, IL15, MCL1, NEFH, PAX5, PKN2**, RGS4, SGK1, SGPL1**, SIAH1, SPARC, SRF, TGFB2, TGFB3, **TPM3**

The biological interpretation of expressional data was performed using Ingenuity Pathway Analysis 7.0 (IPA, Ingenuity Systems Inc., Redwood City, CA). The genes included in the analysis were shown to be differential between F and L and/or between G+ and G- by either microarray or real-time RT-PCR. Genes are presented in alphabetical order for each category. The genes over expressed in muscles with high (F or G+) and low glycogen content (L and G-) are in bold and normal characters, respectively. Genes in italic were differentially regulated between models (F vs. L or G+ vs. G-).

### Validation by real-time RT-PCR of a subset of genes revealed by differential microarray analysis

**Figure 1:**
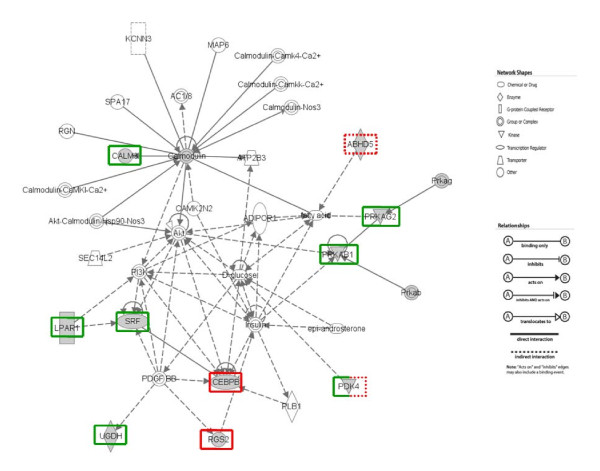
**Network in which several genes identified as differential between Fat (F) and Lean (L) and/or G+ and G- muscles are involved**. The biological interpretation of expression data was performed using Ingenuity Pathway Analysis 7.0 (IPA, Ingenuity Systems Inc., Redwood City, CA). The genes included in the analyses were shown to be differential between F and L and/or G+ and G-. This gene network is involved in several molecular and cellular functions including lipid and carbohydrate metabolism, molecular transport, small molecule biochemistry, and cell morphology. The differential genes surrounded by a dashed line originated from the comparison between F and L birds, and those surrounded by an unbroken line from the comparison between G+ and G- muscles originating from the F2 cross between the F and L lines. The genes over-expressed in muscles with high (F or G+) and low glycogen content (L or G-) are circled in red and green, respectively.

The mRNA levels of 16 genes involved in Skeletal and Muscular System Development and Function, Lipid Metabolism or Carbohydrate Metabolism, and found to be differential on microarray were further quantified by real-time RT-PCR in both models (G+/G- and F/L) (Table 3). The level of 18S rRNA was chosen as reference and confirmed to be invariable. The expression levels (normalized to 18S) of genes were compared between G+ and G- and between F and L (n = 8, same individuals used for microarray analyses) for each of the 16 genes selected. Ratios of gene expression determined by real-time RT-PCR were compared to ratios obtained using microarray analysis (Table 3). Over expression in G+ compared to G- was clearly confirmed for CEBPB and RGS2 (p ≤ 0.05), and suggested for FOXO3 as similar fold-changes reached significance in the microarray analysis but not with real-time RT-PCR. Under expression was confirmed for LPAR1, PDK4, RPS6 and SRF (p ≤ 0.05 or p ≤ 0.10), and suggested for PPP1R2B and UCP3, as similar fold-changes were observed in microarray and RT-PCR studies. None of the differences suggested between F and L chickens by the microarray study could be statistically confirmed by RT-PCR. Slightly higher expression of UGDH (p ≤ 0.10) and lower expression of SRF (p ≤ 0.05) were suggested in F compared to L chickens. It is of note that the genes showed completely different variations between F and L and between G+ and G-. None of the 3 genes over expressed in G+ compared to G- (CEBPB, RGS2, FOXO3) differed between F and L chickens. Similarly, LPAR1 and RPS6, which were under expressed in G+ compared to G-, did not differ between F and L. By contrast to what was observed when comparing G+ to G-, PDK4 was over expressed in F compared to L, suggesting an inverse relationship between muscle glycogen and PDK4 expression in the two models. The observation of higher expression of ABHD5 in G+ compared to G- on microarray prompted real-time RT-PCR measurements in F and L. While the differential expression between G+ and G- was not confirmed, significantly higher expression was observed in F compared to L chickens (p ≤ 0.05). The biological interpretation of the real-time RT-PCR findings highlighted a gene network involved in several molecular and cellular functions, including lipid and carbohydrate metabolism, molecular transport, small molecule biochemistry, and cell morphology (Figure 1).

**Table 3:** Difference in mRNA levels between Fat (F) and Lean (L) and between G+ and G- muscles for genes chosen for quantification by real-time RT-PCR

Symbol	Name	G+/G-		F/L	
		**Microarray**	**qRT-PCR**	**Microarray**	**qRT-PCR**

*Genes screened out from the microarray analyses*					
ABHD5	Abhydrolase domain containing 5	1.367*	0.997	NS	1.527*
ACSL1	Acyl-CoA synthetase long-chain family member 1	1.391*	0.994	NS	0.765
CEBPB	CCAAT/enhancer binding protein (C/EBP), beta	1.491*	3.266**	NS	0.828
ETFA	Electron-transfer-flavoprotein, alpha polypeptide	0.719*	1.036	0.896*	0.896
FOXO3	Forkhead box O3	1.329*	1.400	NS	1.146
LPAR1	Lysophosphatidic acid receptor 1	0.511*	0.472*	NS	1.243
PDK4	Pyruvate dehydrogenase kinase, isozyme 4	0.645*	**0.397†**	ND	**3.007†**
PIK3CD	Phosphoinositide-3-kinase, catalytic, delta polypeptide	1.768*	0.856	NS	1.613
PPP1R12B	Protein phosphatase 1, regulatory (inhibitor) subunit 12B	0.758*	0.776	NS	1.103
RGS2	Regulator of G-protein signaling 2	1.778*	2.433*	1.454*	0.821
RPIA	Ribose 5-phosphate isomerase A	1.380*	0.945	NS	1.352
RPS6	Ribosomal protein S6	0.611*	**0.678†**	NS	1.014
SRF	Serum response factor (c-fos serum response element-binding transcription factor)	0.562*	0.405*	0.734*	0.734
UCP3	Uncoupling protein 3 (mitochondrial, proton carrier)	0.641*	0.601	NS	0.731
UGDH	UDP-glucose dehydrogenase	NS	**0.503†**	1.441*	1.477
UGP2	UDP-glucose pyrophosphorylase 2	1.469*	0.873	NS	0.898

*Candidate genes chosen for targeted analyses by real-time RT-PCR*^§^					
PRKAB1	AMP-activated, beta 1 non-catalytic subunit	ND	**0.603†**	ND	1.315
PRKAG2	AMP-activated, gamma 2 non-catalytic subunit	ND	0.517*	ND	1.129
PHKD	Phosphorylase kinase, delta	ND	0.683*	ND	1.115

For both microarray and real-time RT-PCR analyses, results are expressed as Fat to Lean and G+ to G- ratios of the expression gene.

NS = non-significant; ND = not determined. Statistical significance is indicated as follows: †p value < 0.1, *p value < 0.05, **p value < 0.01.

§ Only genes identified as differentially expressed in at least one model are presented in the Table.

### Differential analysis of candidate genes

The transcript expression of 14 genes (PRKAA1, PRKAA2, PRKAB1, PRKAB2, PRKAG1, PRKAG2, and PRKAG3 encoding the AMP-activated protein kinase α1, α2, β1, β2, γ1, γ2, γ3 subunits, respectively, GYS encoding muscle glycogen synthase, GSK3 encoding glycogen synthase kinase 3, PYG encoding glycogen phosphorylase, and PHKA PHKB, PHKD, and PHKG encoding the glycogen phosphorylase kinase α, β, δ, γ subunits, respectively) directly involved in muscle glycogen turnover was quantified by real-time RT-PCR.

Only 3 of them were significantly differentially expressed between G+ and G- and their expression ratios are presented in Table 3. None of the genes assayed was significantly differentially expressed between F and L chickens. The transcript levels of PRKAB1 and PRKAG2, which encode the AMP-activated protein kinase (AMPK) regulatory β1 and γ2 subunits, respectively was lower in G+ than in G- muscles: The ratio of G+/G- expression was 0.603 (p = 0.07) for PRKAB1 and 0.517 for PRKAG2 (p ≤ 0.05). The PHKD gene, which encodes the δ subunit of phosphorylase kinase (also referred as calmodulin), was also significantly down-regulated (p ≤ 0.05) in G+ compared to G- muscles, with a G+/G- expression ratio of 0.683.

### Transcription factor analysis

**Figure 2:**
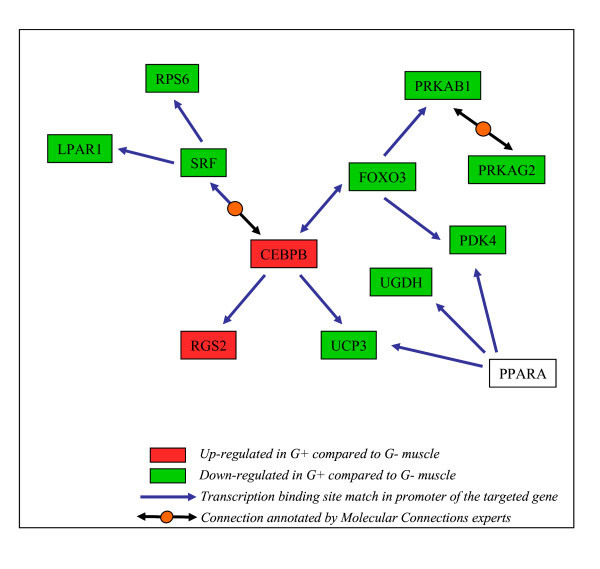
**Summary of interactions between genes differentially expressed between G+ and G- muscles evidenced through a promoter analysis**. Genes highlighted in red and green were up- and down-regulated in G+ compared to G- muscle, respectively. Gene names are indicated in capitals according to Gene Ontology. PPARA, Peroxisome proliferator-activated receptor alpha. Expression of PPARA was not measured in the present study. See Table 3 for other gene names.

Transcription factor analysis highlighted several interrelations between genes whose expression differential was confirmed between G+ and G- animals by real-time RT-PCR (Figure 2). Binding sites for the transcription factor CEBPB were thus reported in the promoters of RGS2, UCP3, SRF, FOXO3. Similarly, promoters of CEBPB, PRKAB1 and PDK4 possess the FOXO3 binding site, and RPS6 and LPAR1 possess binding sites for SRF. UGDH is under the control of PPARA (Peroxisome proliferator-activated receptor alpha) that also activates UCP3 and PDK4.

## Discussion

Few studies have reported global gene expression surveys in the chicken to date. Moreover, our study is the first to relate global gene expression profiles to muscle glycogen content and variations in meat quality. In mammals, especially in cattle and the pig, several microarray studies have investigated global muscle gene expression in relation to sensorial meat attributes such as tenderness, juiciness, flavour and marbling, which are not directly related to muscle glycogen content [15-17]. Recent analyses in the pig helped to link gene expression profiles to variations in meat water loss, this characteristic being strongly related to variations in muscle pH, but without considering glycogen variations in muscle [18]. The transcripts being up-regulated with high drip loss in the pig belong to groups of genes functionally categorized as genes of membrane proteins, signal transduction, cell communication, response to stimulus, and the cytoskeleton. Among genes down-regulated with high drip loss, functional groups of oxidoreductase activity, electron transport, and lipid metabolism were identified.

The originality of our study lies in the models chosen for microarray analyses. As already shown, there is a positive relationship between body fatness and glycogen content in breast muscle in the chicken [11-13]. Comparing divergently selected Fat and Lean chickens, which also differ in muscle glycogen content [13], is therefore relevant to identify mechanisms underlying variations in muscle glycogen directly related to variations in body fatness. It may however not be optimal for distinguishing the mechanisms involved in the control of adiposity and muscle glycogen metabolism. We therefore used chickens generated from a F2 cross between the Fat and Lean lines. In this population, phenotypically extreme individuals with high and low muscle glycogen content (+100% in G+ compared to G-) displayed only limited differences in abdominal fat content (+30% in G+ vs. G-). Being able to dissociate carcass fatness and muscle glycogen content highlighted that, although muscle glycogen metabolism and carcass adiposity are under the control of shared regulation in chicken, they also involve specific pathways. Working on F2 birds also allowed comparison of birds with a more homogeneous genetic background (due to two-generation crossing) while specifically differing in muscle glycogen content. Among the genes found to be expressed in chicken P. major muscle, 197 and 254 transcripts were differentially expressed between F and L and G+ and G-, respectively. Notably, only 12 transcripts, corresponding to 7 known genes, were recorded as differential in both models. With the hypothesis that the G+/G- model was the most powerful to identify genes controlling glycogen metabolism and to rule out the possibility that some of them could have been missed in the F/L comparison, qRTPCR comparisons were conducted on both models and focused on genes linked to glycogen and lipid metabolism and differential between G+ and G-. The results confirm that the differences are indeed specific of the G+/G- model for most genes (Table 3) and even for the genes showing a strong differential expression (CEBPB, LPAR1, RGS2, RPS6) no difference was observed between F and L. Moreover, the results also pointed out that PDK4 and UGDH were inversely regulated in relation to glycogen content in G+/G- compared to F/L. Similarly, we observed significant differences between G+ and G- and not between F and L for three candidate genes (PRKAB1, PRKAG2, PHKD). Altogether, the data further supported that the comparison of extreme animals with high or low glycogen in the F2 population was the most adapted to identify the mechanisms controlling glycogen metabolism in chicken muscle. For this reason we decided to further analyse 12 genes confirmed as differential between G+ and G-.

**Figure 3:**
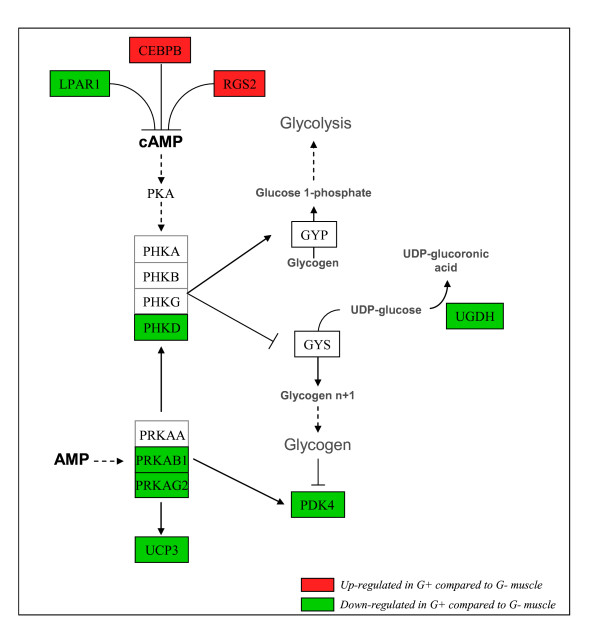
**Summary of changes observed in the expression of genes involved in the regulation of glycogen storage in G+ and G- chickens and putative interactions between them**. Genes highlighted in red and green were up- and down-regulated in G+ compared to G- muscle, respectively. Genes in white boxes were not differentially expressed between G+ and G- muscles. Gene names are indicated in capitals: PHKA, PHKB, PHKG, PHKD, Phosphorylase kinase, alpha, beta, gamma, delta subunit, respectively; PRKAA, PRKAB, PRKAG, AMP-activated protein kinase, alpha, beta, gamma subunit, respectively; PKA, Protein kinase A; GYS, Glycogen synthase; PYG, Glycogen phosphorylase. See Table 3 for other gene names.

A bioinformatic analysis of the promoter sequences of these genes indicated a transcriptional link between 11 of them and suggested a key role for the three transcription factors, CEBPB, FOXO3, SRF as potential regulators of several functional candidates affecting glycogen turnover in the muscle (Figure 2). Figure 3 attempts to summarize how the differences observed at transcript level could impact on glycogen metabolism. The lower expression of UGDH (encoding UDP-glucose dehydrogenase) in the G+ muscle is consistent with reduced conversion of UDP-glucose into UDP-glucuronate, and therefore higher use of glycogen synthesis. PHKD, that encodes the δ subunit of the phosphorylase kinase complex (PHK), was also expressed at lower levels in G+ muscle. This regulatory subunit (also referred to as calmodulin) contains the Ca^2+^-binding site which allows the activation of the phosphorylase kinase complex that both activates glycogenolysis and inhibits glycogen synthesis [19,20]. Its up-regulation in the G- muscle is therefore consistent with increased activity of the PHK complex and a reduced amount of glycogen in muscle. The activity of the PHK complex is under the control of both cAMP-dependent protein kinase (PKA) and AMP-activated protein kinase (AMPK). The nuclear transcription factor CCAAT/enhancer-binding protein beta (CEBPB) and the regulator of G protein signalling 2 (RGS2) are involved in the control of the cellular cAMP level and therefore in the activation of cAMP-dependent protein kinase (PKA) [21-24]. PKA is known to suppress inhibition of the gamma subunit of phosphorylase kinase (PHK) and thereby activate the phosphorylase kinase enzyme complex, which in turn activates glycogen phosphorylase and inhibits glycogen synthase [19,20]. Our observations are therefore consistent with a potential role of CEBPB and RGS2 in the regulation of glycogen levels in chicken skeletal muscle, through the cAMP-dependent PKA pathway. However, the LPAR1 gene (lysophosphatidic acid receptor 1), which exerts a similar effect on cAMP levels, showed different regulation, being down-regulated in G+.

In the muscle of G+ chickens, we observed lower expression of the gene encoding the regulatory γ2 subunit of AMPK (PRKAG2). The AMP-dependence of the AMPK complex is markedly affected by the identity of the γ isoform present, with γ2-containing complexes having a greater response to AMP than those containing γ1 or γ3 [25]. Lower PRKAG2 mRNA levels in G+ muscles could imply a lower response of the AMPK complex to AMP, which in turn would be consistent with greater amounts of glycogen in muscle. Although the levels of AMPK activation were not measured in the present study, a reduced level of AMPK activation was previously reported in F compared to L chickens in conditions where PRKAG2 mRNA levels were lower in F than in L [13]. The PRKAB1 gene encoding the regulatory β1 subunit of AMPK was also expressed at lower levels in G+ muscle, as previously described in [13] when comparing F and L chickens. The AMPK β subunit contains a glycogen-binding site which allows the kinase to act as a glycogen sensor, AMPK activation being inhibited by glycogen [26]. How the changed expression of the β1 regulatory subunit is related to muscle glycogen content remains to be elucidated. While in the former study [13], PRKAB1, PRKAG2 and PHKD were relatively over expressed in the muscles of L compared to F chickens, none of them were found to be differentially expressed between F and L chickens in the present study. One explanation could be that the divergence in abdominal fatness was much more marked (3 fold) between the Fat and Lean birds used in our previous study [13] than in those used in the present study (2 fold), possibly related to the composition of the diet. In fact, the birds used in the first experiment received a diet with a higher crude protein level than those used in the present study (19% instead of 17%) which might explain the lower adiposity differential between Fat and Lean lines reported here. It can be expected that more marked phenotypic differences could be related to more marked differences in gene expression.

A recent study [27] showed that AMPK activation combined with fatty acid administration synergistically induced pyruvate dehydrogenase kinase 4 (PDK4) expression, and in turn decreased cellular glucose oxidation. It can be expected that the preferential expression of the γ2 AMPK subunit (implying potentially greater activation of the AMPK complex as discussed above) in the G- muscles is consistent with the increased expression of PDK4 reported in our study. AMPK activation also increases uncoupling protein 3 (UCP3) expression in muscles in mammals [28,29]. In chicken muscle, stimulation of AMPK is also associated with significant over expression of the avian UCP, the ortholog gene of mammalian UCP3 [30]. The relative down-regulation of the AMPK γ2 subunit was thus consistent with that of UCP3 (or avUCP) reported concomitantly in G+ muscle. As already mentioned, PDK4 was not down-regulated by higher glycogen content in the muscle of the F line, but in contrast, up-regulated, suggesting a strong interaction with lipid metabolism, which could also result from the absence of regulation of AMPK subunits between F and L in the present study which contrasted with earlier results [13].

Several microarray studies conducted in genetically modified mice reported very distinct global gene expression profiles between animals exhibiting high or low glycogen content in muscle [31-33]. There are only a few common genes between those highlighted in our study and those demonstrated to be regulated in such models. This could arise from the fact that our birds were much less divergent in terms of glycogen content than the transgenic mice used previously, and also from the fact that different mechanisms may be involved. In mice, glycogen content was altered by invalidation or overexpression of genes directly controlling glycogen content such as GYS and PRKAG3. The study of Parker et al. (2006) [31] comparing mice lacking [32] or accumulating [33] glycogen in muscle, as a result of glycogen synthase (GYS) inactivation or overexpression, revealed marked differences in expression for a number of enzymes involved in the regulation of glycogen metabolism. Comparison of mutant and knockout mice for PRKAG3 showed that changes in the activity of the γ3 subunit of AMPK were accompanied by coordinated and reciprocal regulation of carbohydrate and lipid metabolism [34]. Indeed, mutation 225Q (corresponding to the RN+ allele gene identified in the pig [7]), which causes accumulation of glycogen in muscle, was associated with a gene expression profile suggesting increased glucose and lipid uptake, oxidative capacity and glycogen synthesis, and resistance of muscle to fatigue. Only slight disturbances in gene expression were observed between G+ and G- chickens, suggesting that the corresponding phenotypes resulted from the additive effects of several genes on the muscle glycogen in the chicken rather than a major effect of a single gene such as the RN gene in the pig [7]. The present study highlighted changes in several candidate genes directly involved in the control of glycogen metabolism such as PHKD, PRKAB1 & G2 and PDK4 and also in genes involved in cAMP signalling such as CEBPB, RGS2 and LPAR1. These transcriptional candidate genes warrant further study in larger populations to correlate their expression with muscle glycogen levels, and to investigate the underlying mechanisms.

## Conclusions

The aim of the study presented here was to identify candidate genes involved in the control of glycogen content in muscle. Studying phenotypically extreme chickens for muscle glycogen content generated from a F2 cross population helped to distinguish mechanisms involved in lipid and carbohydrate metabolism, which are highly related in the chicken. Several genes, related to carbohydrate metabolism or not, were suggested as potentially active in the regulation of muscle glycogen content and hence meat quality in the chicken. A QTL search is in progress on the F2 cross population used in the present study that aims to identify chromosomal regions involved in the control of phenotypes related to chicken meat quality, including muscle glycogen content. This should help to identify both functional and positional genes that could be subsequently included in large scale expression studies to validate the relationship between variations in gene expression and meat quality. These studies should together allow the identification of molecular markers that could be used to select birds with expected muscle and meat properties, and to optimize rearing practices *via *the study of gene regulation.

## Methods

### Animals, Rearing and Slaughtering conditions

Chickens were bred at INRA, UE1295 Pôle d'Expérimentation Avicole de Tours, F-37380 Nouzilly in accordance with European Union Guidelines for animal care and under authorization 37-112 delivered to C. Berri by the French Ministry of Agriculture.

The experimental Fat (F) and Lean (L) lines were generated from a composite meat-type strain of six different origins. F and L lines were divergently selected for abdominal fatness at 9 weeks of age over 7 generations, resulting in a wide difference in carcass fatness [13]. In the first experiment, 8 female chickens from each line were selected from a total of 72 broilers (36 F and 36 L) for further gene expression profile analyses. Within the overall population, the breast muscle glycolytic potential was affected both by line (F > L line, p < 0.01) and sex (female > male, p < 0.01). Although no line/sex interaction was found, only females were included in microarray analysis to rule out any sex effect.

In the second experiment, the gene expression profiles of individuals generated from the F2 population produced from a 2 generation intercross between the founder F and L lines were compared. The F2 population consists of about 600 individuals produced from the cross of 5 F1 sires and 50 F1 dams. The 8 female chickens exhibiting the lowest (G-) and the 8 exhibiting the highest (G+) muscle glycogen content were used for microarray analysis.

In both experimental schemes, the birds were reared up to 9 weeks of age under regular conditions in a conventional poultry house. At 9 weeks of age and after 7 hours of feed withdrawal, the birds were slaughtered and processed at the experimental poultry unit as already described [2].

### Phenotypic traits

Live body weight (BW), abdominal fat percentage and breast yield were measured in addition to ultimate meat pH, meat colour at 24 h post-slaughter, and drip loss after two days of storage at 2°C, as already described [2]. Meat colour was measured by the CIELAB trichromatic system as lightness (L*), redness (a*), and yellowness (b*) values. The glycolytic potential (GP) was determined according to Dalrymple and Hamm [35], from 1 g of muscle tissue collected 15 min. post-slaughter and immediately frozen in liquid nitrogen and calculated as described in Sibut et al. [13].

### RNA isolation

Total RNA was extracted from P. major muscle samples rapidly frozen in liquid nitrogen after death using the Qiagen RNeasy Midi Kit (Qiagen, Courtaboeuf, France), according to the manufacturer's instructions. Concentration and quality of extracted RNA were assessed using a Nanodrop 1000 spectrophotometer (Nanodrop Technology^®^, Wilmington, DE) and a 2100 Bioanalyser (Agilent Technologies, Massy, France), respectively.

### Oligo design and array spotting

The Chicken 20 K Array was obtained from CRB GADIE (INRA Jouy en Josas; http://crb-gadie.inra.fr). The array design has been published in Gene Expression Omnibus with the platform name GPL8199 [36]. The Chicken 20K Oligo set was produced from 20,460 oligonucleotides (60 to 75 nucleotides) designed using the OligoArray 2.0 software against the chicken ENSEMBL transcripts. The transcripts were selected from the chicken genome draft available in December 2004 and extensive matching of the UMIST and DT40 full length EST's with the TIGR clusters (http://chick.umist.ac.uk/). Oligos from a 20K set were arrayed by Operon in 384-well V-bottomed plates (Genetix). Each well contained 1 nmol of oligo. They were resuspended in water on Staccato RapidPlate (Caliper). Spotting was performed on glass slides (Corning, Ultragaps), with 48 Stealth 3 Microspotting pins on Chipwriter (Virtek), with control of humidity (45-50%). After the print run was completed, oligo plates were covered with seals and deep frozen at -20°C in a protected environment. The arrays produced contained exactly the 20,460 oligonucleotides from the original set, 442 buffer spots and 218 unusable oligos (internal control from Opéron). On this array batch, three were used for batch quality control validation: arrays were controlled by SybrGreen to check the presence, intensity and overall shape of the spots and the lack of signal in negative controls (buffer). A lot is considered validated when 95% of observed signals appear where an oligo is expected and if no signal appears in the negative controls.

### Annotation

Because the 20 K oligonucleotide set was defined in 2004 from heterogeneous data sources, the quality of the previously designed oligonucleotides was checked, comparing them with the chromosomes of the 2.1 Washington University assembly of the chicken sequence genome [37]. The comparison was made using NCBI Blast with a 75% similarity threshold over 50 base pairs. The transcripts were then retrieved for each high scoring pair (HSP) corresponding to the location using the Ensembl API (version 3 Ensembl 52). An oligonucleotide had to be in a single gene (even if it was spanning 2 exons) to be selected for further analyses. The corresponding annotations were then retrieved from Ensembl using the Blast HSP coordinates. Among the 20,460 gene-oligonucleotides, 12,907 were identified as aligning with a single coding region in the chicken genome sequence (Version 3.2, February 2009). As an Ensembl gene name and/or a GO biological process term for only 32% of the 12,907 oligo subset were retrieved, it was decided to rely on human orthologs (according to the "one to one" criteria of ENSEMBL annotation) which could be identified for 94% of the 12,907 oligonucleotides, making it possible to retrieve HGNC-HUGO gene symbols for the majority of them (75% of 94% of 12,907). The annotations obtained by a bioinformatics procedure developed by SIGENAE (INRA) are available on the web site: http://www.sigenae.org[37].

### Microarray procedure

#### mRNA labelling and hybridization

Fifty μg of each RNA sample were reverse-transcribed and labelled with Alexa fluorescent-dyes using the SuperScript™ PlusIndirect cDNA Labelling System (Invitrogen, Cergy-Pontoise, France), according to the manufacturer's instructions. A dye-switch procedure was used by labelling F and L or G+ and G- individuals alternately with Alexa 555 green dye and Alexa 647 red dye (Molecular Probes, Invitrogen). After purification, the labelled cDNA samples were quantified using the NanoDrop in order to define the dye concentration and the Frequency Of Incorporation (FOI) which was calculated as follows: FOI (Dye/1000 bases) = Dye (pmol)/cDNA (ng) * 324.5 (pg/mol). According to the manufacturer, the optimal FOI should be between 20 and 50 dye-labelled nucleotides per 1000 nucleotides. The slides were dynamically hybridized at 42°C for 16 h in 30 to 40 μl of buffer (PRONTO!, Corning, Life Sciences ) containing 30 to 50 pmoles of each dye using the SlideBooster (Olympus Advalytix, Germany). Microarrays were then washed with the AdvaWash (Olympus Advalytix, Germany). We finally obtained an initial subset of 8 microarrays for each paired sample from F and L chickens, and a second subset of 8 microarrays for each paired sample from G+ and G-. In all cases, hybridizations were performed with a balanced block design (i.e. half of the samples were labelled with Alexa 555 and the other half with Alexa 647 for each condition). The fluorescence ratio for each gene reflected the relative abundance of the mRNA of interest of either F to L or G+ to G- chickens.

#### Data acquisition

Detection of the fluorescence signals was performed with a laser scanner (GenePix 4000B from Axon Instrument, CA) keeping a constant PMT gain for each channel. Image analysis was performed with GenepixPro 6.0 software (Axon instruments, Inc., Union City, CA) [38]. Raw data files for each array containing all measured values were stored in GenePix files and analysed with the Anapuce 2.0 package (http://cran.r-project.org/web/packages/anapuce/index.html) [39] developed in R language. This package contains functions for the normalization and the analysis of data.

#### Filtering and data normalization

For the normalization step, data were first filtered according to the genepix flag criterion automatically performed by GenepixPro 6.0 [38]. Spots were then discarded in cases of lack of fluorescence homogeneity or overlapping with a contiguous spot. The homogeneity of the background and the fluorescence intensity was systematically checked on each microarray by the boxplot and image plot functions of the R package.

The Alexa 647/Alexa 555 ratio used for analysis was expressed as the log2 of the ratio of median pixel intensity of the two red and green channels. Log2 median ratio values were then normalized on each individual array according to the hypothesis that the majority of gene expressions do not differ between two samples. Normalization was performed by global loess and block effect correction via subtraction of the median per block using the Anapuce 2.0 package [39].

#### Data deposition

The microarray data were deposited in the Gene Expression Omnibus (GEO) public repository http://www.ncbi.nlm.nih.gov/geo The accession numbers for the series are GSE17428 and GSE17445, and the sample series can be retrieved with accession numbers GSM434777 to GSM434784 and GSM435103 to GSM435110. The sample series for each microarray contains the raw data (median signal) of each Alexa637 and Alexa555 channel as well as the normalized data (log2 (Alexa637/Alexa555 ratio)).

#### Data analysis

To identify genes differentially expressed between F and L and G+ and G- chickens we used the DiffAnalysis functions of Anapuce 2.0 under the R statistical environment. Because of the high test numbers, corresponding to the number of genes tested, the raw p value of each gene was adjusted according to the Benjamini-Hochberg method controlling the false discovery rate [40]. Difference in gene expression was judged significant if its adjusted p value was p < 0.05.

### Real-time RT-PCR assay

Five μg of each RNA sample were reverse-transcribed using RNase H^- ^MMLV reverse transcriptase (Superscript II, Invitrogen, Illkirch, France) and random primers (Promega, Charbonnières les Bains, France). A 1/50 or 1/100 dilution of RT reaction, corresponding to 50 or 100 ng RNA equivalent, was then used for real-time quantitative PCR. cDNA samples were mixed with the SYBR Green I qPCR Master Mix Plus (Eurogentec, Angers, France) and specific reverse and forward primers. Primers are described in additional file 4 for genes originating from the microarray analyses and in [13] for candidate genes (PRKAA1, PRKAA2, PRKAB1, PRKAB2, PRKAG1, PRKAG2, and PRKAG3 encoding the AMP-activated protein kinase α1, α2, β1, β2, γ1, γ2, γ3 subunits, respectively, GYS encoding muscle glycogen synthase, GSK3 coding glycogen synthase kinase 3, PYG encoding glycogen phosphorylase, PHKA, PHKB, PHKD, and PHKG encoding the glycogen phosphorylase kinase α, β, δ, γ subunits, respectively). The level of 18 S ribosomal RNA (18S) chosen as a reference was determined with the TaqMan Universal qPCR Master Mix Kit and a pre-developed Taqman assay reagent (Applied Biosystems). Reaction mixtures were incubated in an ABI PRISM 7000 apparatus (Applied Biosystems, Courtaboeuf, France) programmed to conduct one cycle (95°C for 10 min) and 40 cycles (15 s at 95°C and 1 min at 60°C, 62°C or 64°C according to the gene) [13]. For reactions using SYBR Green, a melting curve programme was then performed to check the presence of a single product with a specific melting temperature. Amplification products were checked by electrophoresis and further sequenced. PCR runs for each sample were performed in triplicate. Each PCR run included a no-template control and triplicates of control, i.e. a pool of 12 cDNA samples (i.e. 6 for high and 6 for low GP condition). The calculation of absolute mRNA levels was based on the PCR efficacy and the threshold cycle (CT) deviation of an unknown cDNA versus the control cDNA according to the equation proposed by Pfaffl [41] and as already described [42]. For all genes under study and for 18S, the amplification rates were in the range of 99% to 100% and could be considered as equal to 1. For the same sample, the gene expression level could thus be normalized in relation to the 18S expression level.

### Functional annotation and promoter analysis

The biological interpretation of expressional data was performed using Ingenuity Pathway Analysis 7.0 (IPA, Ingenuity Systems Inc., Redwood City, CA) and Gene Ontology (GO, http://www.geneontology.org/). The genes included in the analyses were shown to be differentially expressed (microarray or real-time RT-PCR) between F and L, G+ and G- or both. For the genes validated as differentially expressed between G+ and G-, analysis of promoters was performed by using the MatInspector and Eldorado applications of Genomatix (http://www.genomatix.de) [43,44].

## Abbreviations

a*: redness; ABHD5: Abhydrolase domain containing 5; ACSL1: Acyl-CoA synthetase long-chain family family member 1; AMPK: AMP-activated protein kinase; b*: yellowness; BW: Body Weight; CEBPB: CCAAT/enhancer binding protein beta; DFD: Dry, Firm and Dry; LPAR1: Endothelial differentiation lysophosphatidic acid G-protein-coupled receptor 2; ETFA: Electron transfer flavoprotein subunit alpha mitochondrial precursor; FDR: False Discovery Rate; GP: Glycolytic Potential; FOI: Frequency of Incorporation; FOXO3: Forkhead protein; GSK3: Glycogen Synthase Kinase 3; GYS: Glycogen Synthase; HGNC: HUGO Gene Nomenclature Committee; HSP: high scoring pair; IPA: Ingenuity Pathway Analysis; L*: lightness; PDK4: Pyruvate dehydrogenase kinase isozyme 4; PHKA: Glycogen Phosphorylase Kinase subunit alpha; PHKB: Glycogen Phosphorylase Kinase subunit beta; PHKD: Glycogen Phosphorylase Kinase subunit delta; PHKG: Glycogen Phosphorylase Kinase subunit gamma; pHu: ultimate pH; PIK3CD: Phosphoinositide-3-kinase catalytic delta polypepetide; PMT: PhotoMultiplier Tube; PPP1R12B: Myosin light chain phosphatase small subunit major isoform; PRKAA1: AMP-activated protein kinase subunit alpha 1; PRKAA2: AMP-activated protein kinase subunit alpha 2; PRKAB1: AMP-activated protein kinase subunit beta 1; PRKAB2: AMP-activated protein kinase subunit beta 2; PRKAG1: AMP-activated protein kinase subunit gamma 1; PRKAG2: AMP-activated protein kinase subunit gamma 2; PRKAG3: AMP-activated protein kinase subunit gamma 3; PYG: Glycogen Phosphorylase; real-time RT-PCR: real-time Reverse Transcription-Polymerase Chain Reaction; SRF: Serum response factor; RGS2: Regulator of G-protein signalling 2; RPIA: Ribose 5-phosphate isomerase A; RPS6: Ribosomal protein; UCP3: Uncoupling protein 3 (mitochondrial proton carrier); UGDH: UDP-glucose dehydrogenase; UGP2: UDP-glucose pyrophosphorylase 2.

## Authors' contributions

VS carried out the gene expression, annotation and statistical analyses and drafted the manuscript. CHA supervised the statistical analyses. CB supervised the study. CB, MJD, and EBD participated in the design of the study, the phenotype data collection and helped to draft the manuscript. SM contributed to the creation of microarrays. All authors read and approved the final manuscript.

## Supplementary Material

Additional file 1 **Genes differentially expressed between Fat and Lean chickens**. Results were expressed as the Fat to Lean ratio of the gene expression. The p value of each gene was adjusted according to the Benjamini-Hochberg method controlling the False Discovery Rate [40]. Difference in gene expression was considered significant if its adjusted p value was p < 0.05. Click here for file

Additional file 2 **Genes differentially expressed between the G+ and G- chickens generated from the F_2_FL population**. Results were expressed as G+ to G- ratio of the gene expression. The p value of each gene was corrected according to the Benjamini-Hochberg method controlling the False Discovery Rate [40]. Difference in gene expression was considered significant if its adjusted p value was p < 0.05. Click here for file

Additional file 3 **List of the common genes that were differentially expressed in the two models (Fat vs. Lean chickens and G+ vs. G- chickens generated from the F**_**2**_**FL population)**. Click here for file

Additional file 4 **Selected real-time RT-PCR primer sequences and accession numbers**. Click here for file
