# Supplementary material for: Identification of differentially expressed genes in chickens differing in muscle glycogen content and meat quality
Source: BMC Genomics. 2011 Feb 16;12:112. doi: 10.1186/1471-2164-12-112 (PMC3047303; doi:10.1186/1471-2164-12-112)
Supplement: Additional file 3 — List of the common genes that were differentially expressed in the two models (Fat vs. Lean chickens and G+ vs. G- chickens generated from the F2FL population). [file 1471-2164-12-112-S3.DOC]

| **ID** | **ENSGAL** | **Name** | **Symbol** |
| --- | --- | --- | --- |
| RIGG01180 | - | - |  |
| RIGG03510 | ENSGALG00000014569 | Patatin-like phospholipase domain-containing protein 2 (EC 3.1.1.3)(Adipose triglyceride lipase) | PNPLA2 |
| RIGG05023 | - | - |  |
| RIGG05099 | ENSGALG00000013255 | Serum response factor (c-fos serum response element-binding transcription factor) | SRF |
| RIGG08973 | - | - |  |
| RIGG09323 | ENSGALG00000014569 | Patatin-like phospholipase domain-containing protein 2 (EC 3.1.1.3)(Adipose triglyceride lipase) | PNPLA2 |
| RIGG11157 | ENSGALG00000002540 | Regulator of G-protein signaling 2 (RGS2) | RGS2 |
| RIGG11393 | ENSGALG00000002921 | LanC-like protein 1 (40 kDa erythrocyte membrane protein)(p40) | LANCL1 |
| RIGG11399 | ENSGALG00000002945 | Transmembrane protein C15orf27 | C15orf27 |
| RIGG13274 | ENSGALG00000006260 | Kelch-like protein 30 | KLHL30 |
| RIGG17320 | ENSGALG00000013255 | Serum response factor (c-fos serum response element-binding transcription factor) | SRF |
| RIGG19676 | ENSGALG00000017328 | P2Y purinoceptor 2 (P2Y2)(P2U purinoceptor 1)(P2U1)(ATP receptor)(Purinergic receptor) | P2RY2 |
