# Supplementary material for: Identification of differentially expressed genes in chickens differing in muscle glycogen content and meat quality
Source: BMC Genomics. 2011 Feb 16;12:112. doi: 10.1186/1471-2164-12-112 (PMC3047303; doi:10.1186/1471-2164-12-112)
Supplement: Additional file 4 — Selected real-time RT-PCR primer sequences and accession numbers. [file 1471-2164-12-112-S4.DOC]

| **Gene symbol** | **Gene name** | **Ensembl or Genebank Accession number** | **Primer sequence (forward /reverse)** |
| --- | --- | --- | --- |
| **ABHD5** | Abhydrolase domain containing 5 | **ENSGALG00000011510** | TTTTACCAGGGCTGGGGAATGG  AATGCACTAATCTGCTGTGGGTG |
| **ACSL1** | Acyl-CoA synthetase long-chain family member 1 | **ENSGALG00000010628** | CCTTCGCTGCATTAACACAATTCC  CCACATTCATCATGGGGAAAAC |
| **CEBPB** | CCAAT/enhancer binding protein (C/EBP), beta | ENSGALG00000008014 | GCAAGAACAAGCCCAAGAAGTG  CAAGACTTTGTGCTGCGTCTCC |
| **LPAR1** | Lysophosphatidic acid receptor 1 | ENSGALG00000015729 | GGAATCGGGACACGATGATGAG  CGGTAGGAGTAGATGATGGGGTTC |
| **ETFA** | Electron-transfer-flavoprotein, alpha polypeptide | ENSGALG00000002925 | TGAACAAACAGAGCAGCAACAGG  CAGATGGCAGCTTCAGGAACAC |
| **FOXO3** | Forkhead box O3 | ENSGALG00000015297 | CGTTGTCAGTCTGAATGTGGGG  GACAGCAGATTTGGCAAAGGG |
| **PDK4** | Pyruvate dehydrogenase kinase, isozyme 4 | ENSGALG00000009700 | TGACTGGTGCATCCCAAGTAAAG  GGAAGAATTTGCCTGTTTGGAGG |
| **PIK3CD** | Phosphoinositide-3-kinase, catalytic, delta polypeptide | ENSGALG00000002583 | GGCTCTGGGTAAAACAGATGAAGAG  GATCATTGGATTCAGTGCAAGTGG |
| **PPP1R12B** | Protein phosphatase 1, regulatory (inhibitor) subunit 12B | ENSGALG00000000336 | TCTGGGGAATGGAAACGAGG  TCTGCTGGATGAAAGGACTCTGAC |
| **RGS2** | Regulator of G-protein signaling 2, 24kDa | ENSGALG00000002540 | CTGACGCTGAAGGCAAAGAAAATC  CAGAAACCGTGGGTACGAGTTG |
| **RPIA** | Ribose 5-phosphate isomerase A | ENSGALG00000015960 | GAAGGATAGAGTGGAATGCAAAGG  CACGAGAATCATGCAAAGCACC |
| **RPS6** | Ribosomal protein S6 | ENSGALG00000015082 | GTGAGAAGGATATTCCTGGGCTG  CAGAGGTTTCCTCACAACATACTGG |
| **SRF** | Serum response factor (c-fos serum response element-binding transcription factor) | ENSGALG00000013255 | **ACAGCAGCACTGACCTTACTCAGAC**  **TCTGCGAGAAAGCATTGAGGAC** |
| **UCP3** | Uncoupling protein 3 (mitochondrial, proton carrier) | ENSGALG00000017316 | CTATGGGATGAGAGGGACCAAAG  GGAGAACCTCAACACGTTCCTTC |
| **UGDH** | UDP-glucose dehydrogenase | ENSGALG00000014320 | CCTATGGGATGGGAAAAGGGAG  GGGTTAGACAGCACCTGCAAATC |
| **UGP2** | UDP-glucose pyrophosphorylase 2 | ENSGALG00000008855 | CTTCACGAAGGTCCAGGATTACC  TCTTCCCAAATGTAACATCGCC |
